# Editorial: Monogenic vs. Oligogenic Reclassification

**DOI:** 10.3389/fgene.2021.821591

**Published:** 2021-12-13

**Authors:** Olfa Messaoud, Atanu Kumar Dutta, Mario Reynaldo Cornejo-Olivas, Zahurul A. Bhuiyan

**Affiliations:** ^1^ Biomedical Genomics and Oncogenetics Laboratory, Institut Pasteur de Tunis, University Tunis El Manar, Tunis, Tunisia; ^2^ Department of Biochemistry, All India Institute of Medical Sciences, Kalyani, India; ^3^ Neurogenetics Research Center, Instituto Nacional de Ciencias Neurologica, Lima, Peru; ^4^ Center for Global Health, Universidad Peruana Cayetano Heredia, Lima, Peru; ^5^ Unité de Recherche Cardiogénétique, Service de Médecine Génétique, Centre Hospitalier Universitaire Vaudois (CHUV), Lausanne, Switzerland

**Keywords:** monogenic, digenic, oligogenic, bilocus combination, additive risk

During the pre-genomic era, the boundary between monogenic and polygenic diseases was so clear that we assigned almost all rare diseases to the monogenic class and roughly all common diseases to the multigenic class. Nowadays, this classification is no longer applicable since there is a growing list of diseases associated with multigenic influence compounded by other environmental or physical components. This is likely the case for many complex disorders such as endocrine, cardiac, obesity, ciliopathy, and neurodevelopmental disorders. Examples of oligogenic endocrine disorders include Congenital Hypothyroidism, Congenital Hypogonadotropic Hypogonadism (CHH) and Disorders of Sex Development (Camats et al., 2020).

Mkaouar et al. in their article, studied the role of oligogenic inheritance of *PROKR2* mutations in CHH based on genetic profiles of two different families harbouring pathogenic variants in the *PROKR2* gene, including asymptomatic homozygous carriers. Their findings illustrate a digenic inheritance involving *PROKR2*-*CCDC141* and *DUSP6*-*SEMA7A* and hence confer more evidence that homozygous loss-of-function genetic variations are insufficient to cause Kallman Syndrome; having oligogenic mechanisms as the most likely responsible of CHH.

Unlike CHH, Skeletal Dysplasias are often genetically well-characterized and involving generally a monogenic inheritance. However, this is not the case for a minority of cases as reported by Costantini et al.who describes an 11-year-old Finnish girl with a phenotype similar to Odontochondrodysplasia (ODCD) and a family history of a foetus lost due to severe skeletal dysplasia. By conducting an oligogenic inheritance approach in analyzing exome sequencing data, the authors identified five pathogenic variants in different skeletal dysplasia genes (*TRIP11, FKBP10*, *TBX5*, *NEK1*, and *NBAS*). The foetus was found homozygous for the *TRIP11* mutation, hence confirming the diagnosis of Achondrogenesis type IA. This study illustrates once more the cumulative effect of pathogenic variants in multiple genes as a mechanism of oligogenism for bone development-related disorders and underlines the importance of screening for all phenotype-related variants since two different disease entities could be observed in one family (Mishra et al., 2020). Likewise, we could also perceive variations in disease severity when multiple mutations are found, either in the same gene or in different genes that are implicated in the same disease. It is also well known that some common SNPs, usually benign, could also modify the penetrance of the disease when segregated in combination with the disease causal mutation (Crotti et al., 2005).

Oligogenic disorders could lead to ciliopathies as reported by Dallali et al. who described a Tunisian patient experiencing learning and language difficulties, short stature and Brachydactyly. Whole exome sequencing revealed five heterozygous variants in different genes including *BBS1*, *BBS4*, *BBS8*, *MKS1*, and *CEP290*. All encode proteins related to cilium biogenesis. Authors showed in-silico evidence of potential cumulative synergic effects of these variants.

Several examples of oligogenic diseases could be also drawn from inherited cardiac diseases. In his review, Wallace et al. reviewed the genetic basis of sinoatrial node dysfunction (SND). He underlined that this cardiac pacemaking dysfunction requires a better understanding of the underlying mechanisms since most of SND-related genes, exhibit multiple unrelated pleiotropy and phenotypic traits.

In line with this genetic diseases mechanism thinking, Early Myocardial Infarction and Stroke could have a monogenic basis, caused either by loss of function mutations in *LDLR* and *ApoB* genes, or gain of function mutations in *PCSK9* gene (Cohen et al., 2005; Gidding et al., 2015). Nonetheless, a recent study elucidated that Early Myocardial Infarction could also be due to cumulative effects of numerous common genetic variants based on polygenic risk score assessment. So genetics is no more limited to identifying disease causing rare variants, it’s also gaining access into the domain of common diseases (Khera et al., 2019).

**FIGURE 1:**
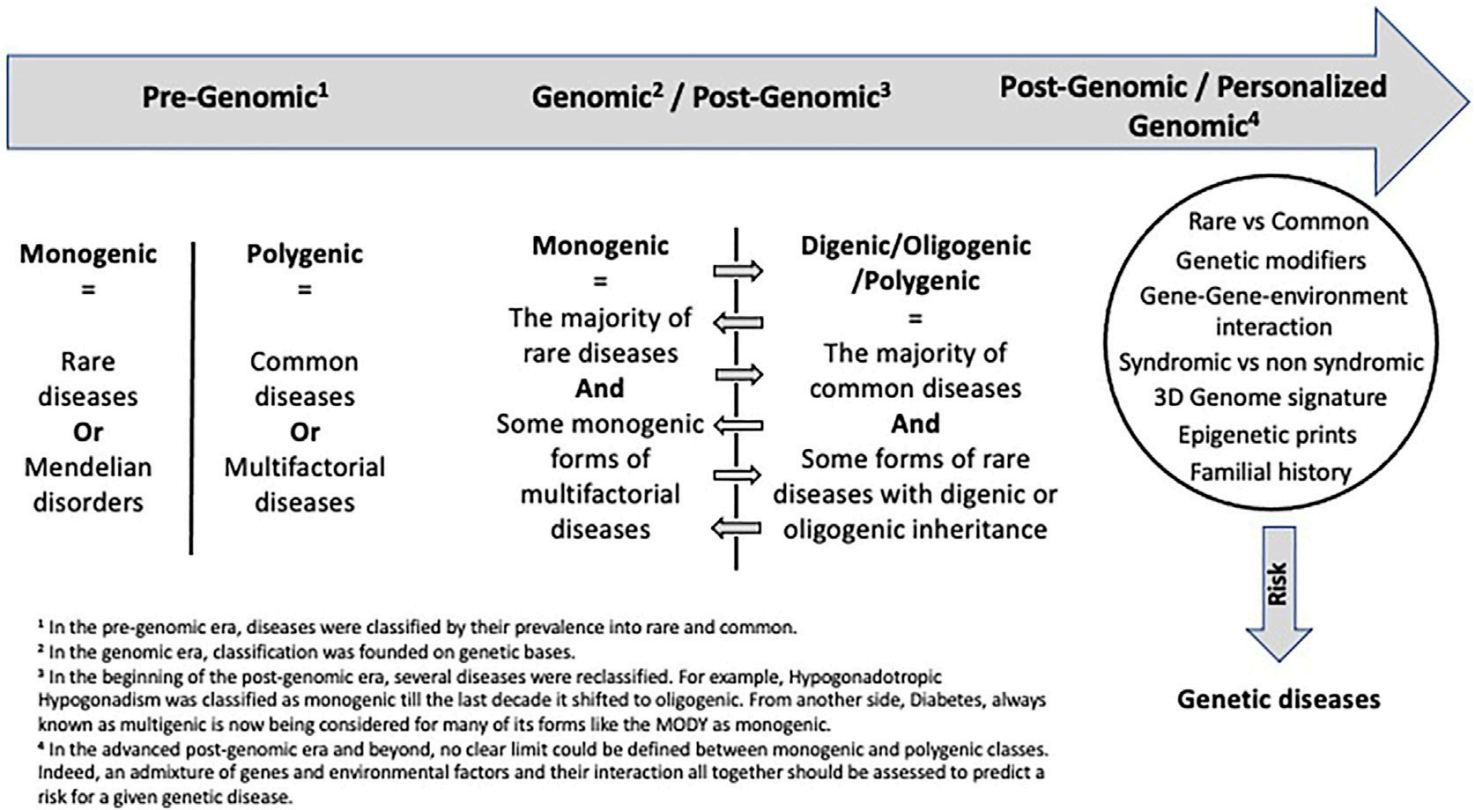
Evolution of disease classification through genomic time.

Through genomic time, monogenic vs. oligogenic diseases have been reclassified (Figure 1). This classification evolution is mainly resulting from technological advances. Indeed, the beginning of the post-genomic era was essentially marked by an increased accessibility and affordability of Next Generation Sequencing (NGS) technologies, allowing the discovery of the hidden genetic components for several inherited disorders and unravelling the complex genetic mechanisms behind them. Since then, we have observed a gradual shift from the monogenic model to the oligogenic one. As of October 2021, 211 OMIM entries include the term “digenic” and 84 include “oligogenic,” with more and more disorders being reclassified as oligogenic.

However, despite the significant advances in this new paradigm, other intriguing aspects remain to be explored in the field of oligogenic disorders and complex inheritance, not only in terms of identifying the causative genes but also in understanding 1) how variants located in different genes interact together to confer a cumulative risk for a given disease, 2) how common genetic variants affect the outcome of several common diseases. The most important point to start with is to define the nature of this epistatic interaction: Is it a true digenic inheritance, a pseudo-digenic inheritance, do one of the variants acts as a genetic modifier or is it just a case of co-inheritance (Deltas, 2018)?

We envisage that future of genomic medicine lies into understanding the role and eventual clinical use of common genetic variants into the onset and outcome of various common diseases. Perhaps, only naked DNA variants are not sufficient to interpret such disease modifying effects, studies into methylation, metabolomics, proteomics, and many other modifying agents, which in combination might lead us to understand into the deeper disease mechanisms and treat the common diseases in an individualistic approach, which we foresee to integrate in our future personalized genomic medicine.
